# Enhancing diagnostic precision for rare diseases using case-based reasoning

**DOI:** 10.1093/jamia/ocaf092

**Published:** 2025-06-17

**Authors:** Richard Noll, Alexandra Berger, Carlo Facchinello, Katharina Stratmann, Jannik Schaaf, Holger Storf

**Affiliations:** Institute of Medical Informatics, Goethe University Frankfurt, University Medicine, 60590 Frankfurt am Main, Germany; Frankfurt Reference Centre for Rare Diseases, University Medicine, Goethe University Frankfurt, 60596 Frankfurt am Main, Germany; Academic Teaching Practice of the Heinrich-Heine-University Düsseldorf, 40885 Ratingen, Germany; Medical Clinic 1, Goethe University Frankfurt, University Medicine, 60596 Frankfurt am Main, Germany; Institute of Medical Informatics, Goethe University Frankfurt, University Medicine, 60590 Frankfurt am Main, Germany; Institute of Medical Informatics, Goethe University Frankfurt, University Medicine, 60590 Frankfurt am Main, Germany

**Keywords:** case-based reasoning, rare diseases, diagnostic techniques and procedures, medical informatics, interdisciplinary research

## Abstract

**Objective:**

This study aims to enhance the diagnostic process for rare diseases using case-based reasoning (CBR). CBR compares new cases with historical data, utilizing both structured and unstructured clinical data.

**Materials and Methods:**

The study uses a dataset of 4295 patient cases from the University Hospital Frankfurt. Data were standardized using the OMOP Common Data Model. Three methods—TF, TF-IDF, and TF-IDF with semantic vector embeddings—were employed to represent patient records. Similarity search effectiveness was evaluated using cross-validation to assess diagnostic precision. High-weighted concepts were rated by medical experts for relevance. Additionally, the impact of different levels of ICD-10 code granularity on prediction outcomes was analyzed.

**Results:**

The TF-IDF method showed a high degree of precision, with an average positive predictive value of 91% in the 10 most similar cases. The differences between the methods were not statistically significant. The expert evaluation rated the medical relevance of high-weighted concepts as moderate. The granularity of ICD-10 coding significantly influences the precision of predictions, with more granular codes showing decreased precision.

**Discussion:**

The methods effectively handle data from multiple medical specialties, suggesting broad applicability. The use of broader ICD-10 codes with high precision in prediction could improve initial diagnostic guidance. The use of Explainable AI could enhance diagnostic transparency, leading to better patient outcomes. Limitations include standardization issues and the need for more comprehensive lab value integration.

**Conclusion:**

While CBR shows promise for rare disease diagnostics, its utility depends on the specific needs of the decision support system and its intended clinical application.

## Background and significance

Globally, approximately 10 000 distinct rare diseases and disorders affect more than 300-400 million people, presenting substantial challenges in medical diagnostics.^1^^,^^2^ The infrequent occurrence can result in primary care physicians failing to consider them when making a diagnosis. The diffuse and complex symptomatology associated with these medical conditions often leads to diagnostic ambiguity. In addition, there are only a few specialized centers for rare diseases, whose waiting times are long, which can often only accept patient cases after a strict selection process. In rural areas, few services and physicians can guarantee appropriate further care.^3^

The fundamental challenge in diagnosing rare and unclear diseases lies in the lack of comprehensive data and previous case studies, which conventional diagnostic methodologies rely on. This gap often results in delayed or inaccurate diagnoses, which can have a detrimental impact on patient care. A considerable number of patients with rare diseases remain undiagnosed for an extended period, even after the use of advanced diagnostic techniques such as gene panels and exome sequencing. Many patients ultimately die without an accurate diagnosis.^2^

The application of computer-aided diagnostic methods, such as supervised and unsupervised machine learning, is frequently constrained by the unavailability of dependable “gold standard” diagnoses upon which to base its learning. Consequently, such tools frequently operate on noisy labels (“silver standard”) or focus on individual rare diseases.^4^^,^^5^ Patient-similarity-based models offer diagnostic decision support that is more accurate, generalizable, and interpretable. Furthermore, they can work on heterogeneous and incomplete data and typically require less extensive datasets than traditional machine learning models do, making them more feasible in data-limited scenarios.^6^ The current body of literature highlights several methodologies that aim to increase diagnostic accuracy and precision through the comparison of standardized case concepts and clinical features among patients.^6^^,^^7^ Approaches such as those implemented by Garcelon et al. based on Unified Medical Language System (UMLS) concepts and a vector space model (VSM) with a joint clinical data warehouse of 3 million electronic health records (EHRs) serve to illustrate the potential of case-based strategies in medical diagnostics for rare diseases.^7^ The advantage of this strategy—in comparison with machine learning approaches—is the transparency of the output for the subsequent decision maker. This is because the prediction is not based on rules learned in a black box, but on representable and explainable similarity metrics.^8^

In this study, case-based reasoning (CBR) is proposed to improve the diagnostic process for rare diseases. CBR seeks to draw parallels between new patient cases and historical data.^8^ This study includes extracting information from structured and unstructured data. Rosenbloom et al. demonstrated the importance of information extraction from unstructured clinical narratives for most clinical applications.^9^ By coding comprehensive disease progressions using standardized medical vocabularies, 3 methods for similarity search were investigated.

In previous studies, the challenges associated with the standardization of medical data have been insufficiently addressed. Although the utilization of standardized vocabularies, such as Logical Observation Identifiers Names and Codes (LOINC),^10^ is vital for guaranteeing uniformity in data representation, the intricacy and variability intrinsic to these systems frequently result in issues such as inconsistencies, specialized regulations, linguistic variations, and ambiguous abbreviations.^11–13^ This study aims to address these gaps by introducing an approach that goes beyond traditional statistical methods. By incorporating a semantic component through medical terminology embeddings enriched with domain-specific knowledge, this research aims to capture the contextual meaning of terms more effectively.^14–16^ In addition, this research takes into account critical elements such as the inclusion of reference ranges in laboratory results, which can significantly improve the reliability of diagnostic procedures.

## Objective

The primary objective of this research is to evaluate the effectiveness of the proposed methods, with a particular focus on their utility in the context of rare diseases collected at the University Hospital in Frankfurt, Germany, from 2015 to 2022. The effectiveness of a method is evaluated by measuring its ability to identify the correct diagnosis within the *k* most similar cases using metrics such as precision, success rate and empirical standard deviation. These metrics, in conjunction with expert evaluation, consideration of diagnostic granularity, and acknowledgment of limitations, are used to assess the system's utility in supporting clinical decision-making for rare diseases. This research contributes to the theoretical understanding of CBR in medical diagnostics and has the potential to advance the field of practical approaches to diagnosing rare diseases.

## Materials and methods

A CBR framework is employed to enhance the diagnostic process for rare diseases. The study design involves a comparative analysis of 3 similarity search methods applied to patient cases of the following data source.

### Data sources

The foundation of this study is a dataset that includes patient cases from 3 medical specialties: Gastroenterology, Pulmonology, and Endocrinology. These cases were collected at the University Hospital in Frankfurt. A total of 4295 patient cases were included, each with at least one primary diagnosis from Table 1, coded via the International Statistical Classification of Diseases and Related Health Problems, 10th rev., German Modification (ICD-10-GM),^17^ and various associated attributes, including medical conditions, measurements (ie, laboratory tests, vital signs, quantitative findings), observations (ie, symptoms, lifestyle facts, medical history), and gender. Gender-specific differences, which correspond to biological sex differences, are evident in pulmonological and endocrinological diseases.^18^^,^^19^ The primary diagnosis is the main medical condition identified during an inpatient visit to a healthcare facility in Germany, which is primarily responsible for the patient’s need for treatment or evaluation.

**Table 1.** Diseases from the 3 medical specialties and their frequencies in the dataset for 4295 patients.

Specialty	Primary diagnosis	ICD-10-GM	Frequency	Percentage (%)
Gastroenterological	Acute Hepatitis A	B15.-	28	0.53
	Acute Hepatitis B	B16.-	39	0.74
	Other acute viral hepatitis	B17.-	76	1.44
	Chronic viral hepatitis	B18.-	130	2.46
	Wilson's disease	E83.0	7	0.13
	Hemochromatosis	E83.1	24	0.45
	Alpha-1-antitrypsin deficiency in adults	E88.0	4	0.08
Pneumological	Bronchial carcinoma	C34.-	2584	48.83
	Tuberculosis	A15.-	221	4.18
	Sarcoidosis	D86.-	345	6.52
	Viral infection	J11.-	74	1.40
Endocrinological	Clinical hyperthyroidism	E05.-	1749	33.05
	Thyroiditis de Quervain	E06.1	11	0.21
			**5292**	100

One patient can have several of these primary diagnoses. If no digit is specified after the dot, eg, B15.-, in the ICD-10-GM, all available digits are included.

The admission and discharge dates of the patients ranged from January 1, 2015 to December 31, 2022. The patients were older than 18 years at the time of admission. Full or partial inpatient hospital service was provided. The selection of these primary diagnoses was made with 2 considerations in mind, as discussed by an interdisciplinary group.^20^ First, there is a focus on clinical expertise in these areas at the University Hospital in Frankfurt. Second, the available data in these areas are of high quality, comprising completeness, accuracy, and reliability.

In the European Union, a rare disease is defined as affecting fewer than 1 in 2000 people.^1^ Although not all selected diagnoses necessarily fall under this definition of rare diseases, diagnoses that are often ambiguous and therefore present physicians with a challenge comparable to that of rare diseases were selected by clinical experts at the University Hospital in Frankfurt and included in the selection.

### Data translation

To facilitate communication between different computational infrastructures, it is common practice to use standards such as Fast Healthcare Interoperability Resources (FHIR). FHIR is an HL7 standard designed to enable the exchange of healthcare information, promoting interoperability and the integration of data across different systems.^21^ The FHIR-based core dataset definitions of the Medical Informatics Initiative (MII) in Germany were used for the data transfer of the cases. The MII Core Dataset is a standardized dataset agreed upon by all MII consortia and university medical centers in Germany.^22^

The standardization of patient data was crucial to ensure uniformity. The extracted data were converted from FHIR into the Observational Medical Outcomes Partnership (OMOP) Common Data Model (CDM) via an Extract-Transform-Load (ETL) pipeline, which enables systematic analysis and comparison.^20^ The OMOP CDM is an internationally established research data repository of the Observational Health Data Science and Informatics (OHDSI) community for the harmonization and standardization of clinical observational data for research purposes.^23^^,^^24^ The patient attributes were mapped to standard vocabularies within the OMOP CDM to ensure consistent data representation. To ensure compliance with the FAIR principles,^25^ the mappings are stored in a transition database.

Natural language processing (NLP) was used for non-structured and non-standardized narrative reports. The Averbis Health Discovery Tool^26^ extracted symptoms from German physician notes and mapped them to the Human Phenotype Ontology (HPO) for standardized representation.^27^ A semi-automated translation process was used to map the German terms to the English HPO.^28^^,^^29^ The HPO is a highly regarded resource within the rare disease community for differential diagnosis.^27^

After standardization, each case in the OMOP CDM consists of several medical concepts with unique identifiers. Medical concepts are used to describe information in a patient's EHR. The following rules were employed to prepare the data for vector representation:

1. The concepts for each patient were compiled into a single patient record.
2. To ensure clarity across different vocabularies and to define a concept c, the vocabulary name was concatenated with the concept identifier, such as “SNOMEDCT|235856003,” which represents the Systematized Nomenclature of Medicine Clinical Terms (SNOMED CT)^30^ and the identifier (235856003: “Disease of liver”).
3. For laboratory values, the relationship to the reference range was included if available, such as “LOINC|1742-6|above,” which represents the LOINC vocabulary,^10^ the identifier (1742-6: “Alanine aminotransferase”), and the relation to the reference range (“above”). The relation specifications could be “below,” “within,” or “above,” indicating that the results were below, within, or above the reference range, respectively.
4. The concept name cn (eg, “Disease of liver”) was stored for the semantic analysis.
5. The primary diagnoses and directly indicative concepts, such as the LOINC value “91071-1: Hepatitis E virus RNA [Presence] in Stool by NAA with probe detection,” were excluded from the records. The primary diagnosis serves as the benchmark for validation.
6. Only patients with at least 2 concepts in their record were considered.

### Data analysis

In this study, vector representations are employed for patient records, integrating statistical and semantic methods. The weight calculation process comprises 3 stages: (1) calculating term frequency (TF) to enumerate concept occurrences, (2) applying term frequency-inverse document frequency (TF-IDF) to adjust these counts by the rarity of each concept across all cases, and (3) enhancing TF-IDF vectors with semantic embeddings to capture contextual meanings. The resulting weights are presented in the matrix shown in Table 2, with rows representing patients and columns representing concepts.

**Table 2.** The weight matrix for patient cases and medical concepts.

	*c_j_*	*…*	*c_M_*

*p_i_*	*w_ij_*	*…*	*w_iM_*
*…*	*…*	*…*	*…*
*p_N_*	*w_Nj_*	*…*	*w_NM_*

Each row corresponds to a patient record (*p_i_*), and each column corresponds to a medical concept (*c_j_*). The cell values *w_ij_* denote the weight of concept *c_j_* in patient record *p_i_*. In this context, *N* represents the total number of patient records, whereas *M* corresponds to the total number of concepts.

#### TF weighting

Each case's data are extracted and parsed to identify concepts $c_{j}$, which are then used to form a distinct representation of each patient record $p_{i}$^31^:


(1) $$TF(c_{j},p_{i})=\text{Number}\;\text{of}\;\text{times}\;\text{concept}\;c_{j}\;\text{appears}\;\text{in}\;\text{patient}\;\text{record}\;p_{i}.$$

#### TF-IDF weighting

Inverse document frequency (IDF) assesses the significance of a concept across a dataset of patient records. It is inversely proportional to the frequency of the concept across the records, calculated as the logarithm of the ratio of the total number of patient records (N) to the number of records containing the concept:


(2) $$\mathrm{IDF}(c_{j},N)=\log(\frac{1+N}{1+\text{Number}\;\text{of}\;\text{patient}\;\text{records}\;\text{containing}\;\text{concept}\;c_{j}})+1.$$

The addition of “1” to the IDF in eqn (2) ensures that terms with zero IDF, or those that occur in all the documents in the training set, are not entirely disregarded.^32^ The TF-IDF score is then the product of these 2 statistics,^33^ providing a weight for each concept:


(3) $$TF\cdot \mathrm{IDF}(c_{j},p,N)=TF(c_{j},p_{i})\times \mathrm{IDF}(c_{j},N).$$

This formula emphasizes the relevance of specific concepts within individual patient records, particularly those unique to certain cases.

#### TF-IDF with semantic vector embeddings

Semantic embeddings are vector representations of words or phrases that capture their meanings based on context. These embeddings are generated using deep learning models such as transformers, which are trained on large corpora.^34^ The primary goal is to map words with similar meanings to nearby points in a high-dimensional vector space. Given a concept name cn, an embedding model M generates a vector $\vec{e}\in R^{d}$, where d is the dimensionality of the embedding space.

A concept map is used to cluster semantically similar terms based on their embeddings,^14^ facilitating the normalization of terms in a dataset. The process involves several steps:

- **Embedding calculation for concepts**: For each unique concept name ${cn}_{i}\;\mathrm{in}\;\mathrm{the}\;\mathrm{dataset},\;\mathrm{its}\;\mathrm{embedding}\;{\vec{e}}_{c_{i}}$ is computed.
  (4) $${\vec{e}}_{c_{i}}=M({cn}_{i}).$$
- **Similarity matrix construction**: A similarity matrix $S_{ij}\;\mathrm{is}\;\mathrm{constructed},\;\mathrm{where}\;\mathrm{each}\;\mathrm{entry}\;S_{ij}\;\mathrm{represents}\;\mathrm{the}\;\mathrm{cosine}\;\mathrm{similarity}\;\mathrm{between}\;\mathrm{embeddings}\;e_{c_{i}}\;\mathrm{and}\;e_{c_{j}}$.
  (5) $$S_{ij}=\text{cosine}\_\text{similarity}({\vec{e}}_{c_{i}},{\vec{e}}_{c_{j}}).$$
- **Thresholding for concept mapping**: A similarity threshold θ is defined. If $S_{ij}\;$≥ θ, ${cn}_{i}\;\mathrm{and}\;{cn}_{j}\;\mathrm{are}\mathrm{considered}\;\mathrm{similar}.\;\mathrm{These}\;\mathrm{concepts}\;\mathrm{are}\;\mathrm{mapped}\;\mathrm{to}$ a representative concept. This can be achieved through a mapping function *f* such that:
  (6) $$f({cn}_{i})=f({cn}_{j})\;\text{if}\;S_{ij}\geq \theta .$$
*Example:* For θ = 0.9, if$\;S_{ij}=\text{cosine\_similarity}{\;\vec{e}}_{\text{cough}},{\vec{e}}_{\text{coughing}}\geq 0.9$, then f(cough)=f(coughing)=cough.
- **Application of the concept map**: The dataset is normalized by replacing each concept with its representative concept as defined by the mapping function f.

These semantic vectors utilize embeddings pretrained on medical terminology to encapsulate domain-specific knowledge. Contrastive learning on knowledge graphs for Cross-lingual Medical Term Representation (CODER) was used, to establish similarities between terms.^14^ The CODER system has been developed for the normalization of medical terminology, with the objective of providing close vector representations and being trained via contrastive learning on the UMLS.^35^ The semantic embedding vector ${\vec{e}}_{c_{i}}$ has d = 768.

### Evaluation

Cosine similarity was used to evaluate the similarity between the vector of an index patient (${\vec{p}}_{\mathrm{index}}$) and that of another patient (${\vec{p}}_{i}$). This measure has been broadly used to compute similarity in the biomedical domain.^36–38^ It calculates the cosine of the angle between 2 vectors in the VSM, providing insight into their orientation:


(7) $$\cos\left(\theta_{\mathrm{index},\;i}\right)=\frac{{\vec{p}}_{\mathrm{index}\;}\cdot \;{\vec{p}}_{i}}{||{\vec{p}}_{\mathrm{index}}||\times ||{\vec{p}}_{i}||\;},$$

where ‘⋅' denotes the dot product and ‘‖$\vec{p}$‖' indicates the *L2* norm of each vector, which entails scaling each vector so that the sum of the squares of its elements is equal to 1. This ensures that each vector has a unit length and facilitates the calculation of similarity. High cosine similarity scores indicate significant alignment between patient records. The value ranges from −1 (exactly opposite) to +1 (the same), with 0 indicating orthogonality (no similarity).

**Figure 1.**
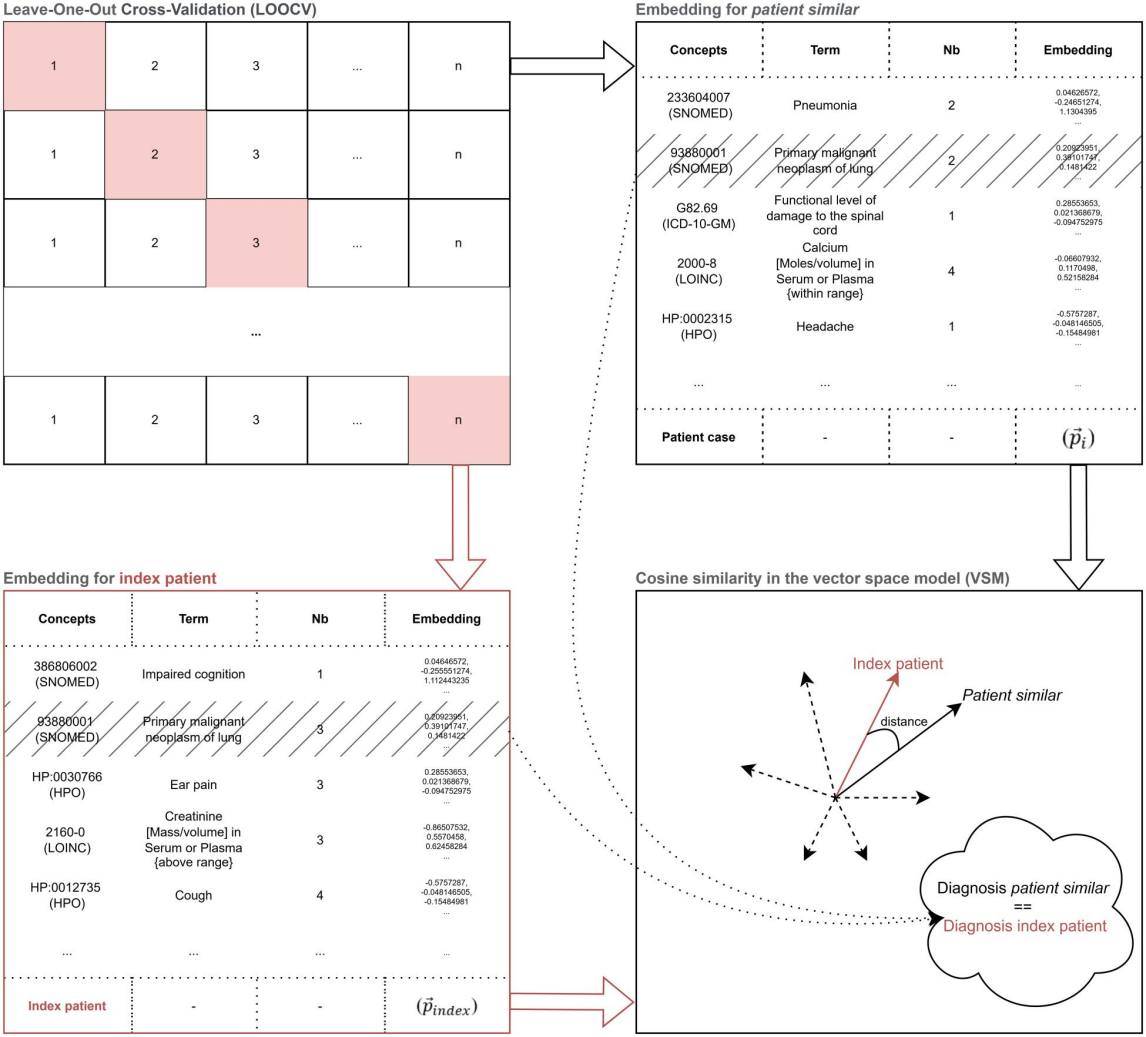
The LOOCV procedure involves selecting a single patient from the database as the validation set (index patient). The primary diagnoses are removed and later compared for validation after the *k* most similar patients have been identified. The identifiers with the vocabulary “Concepts,” the name “Term,” the frequency “Nb,” and the semantic vector “Embedding” of the concepts are displayed.

To ensure a robust assessment and generalizability of the 3 different variants of vector representation, Leave-One-Out Cross-Validation (LOOCV) was implemented, as illustrated in Figure 1. LOOCV is a form of cross-validation where one data point is used as the validation set (index patient), and the remaining data points constitute the training set. This process is repeated such that each data point serves as the validation set exactly once.^39^ The decision to employ LOOCV was driven primarily by both the modest dataset size and the heterogeneous distribution of the specific case groups.

The analysis determined the true positives (TPs)—records that were among the k most similar patients and shared at least one primary diagnosis with the index patient. False positives (FPs) were identified as records that, despite being among the k most similar, did not share a primary diagnosis with the index patient.

The average precision was calculated for the top k patients, where k was set to 1, 3, 5, and 10. The formula used for calculating the average precision is as follows:


(8) $$\text{Precision}(k)=\frac{1}{N}\sum_{i=1}^{N}\frac{\sum_{j=1}^{k}I({\text{TP}}_{ij})}{k},$$

where N is the total number of patient records, and I(…) is the indicator function that equals 1 if the *j*-th prediction for the *i*-th index patient is a TP and 0 otherwise. The sum $\sum_{j=1}^{k}I({\text{TP}}_{ij})\;$counts the number of TPs among the top *k* predictions for each index patient.

A precision of 1 signifies that each patient predicted by the model as similar (top k) has one of the primary diagnoses of the index patient. A precision value approaching zero indicates a high prevalence of FPs, which suggests that the model's ability to identify similar patients may be limited.

The empirical standard deviation $s_{k}$ of the precision for the top *k* similar patients is calculated as


(9) $$s_{k}=\sqrt{\frac{1}{N}\sum_{i=1}^{N}{\left(\frac{\sum_{j=1}^{k}I({\text{TP}}_{ij})}{k}-\frac{1}{N}\sum_{i=1}^{N}\frac{\sum_{j=1}^{k}I({\text{TP}}_{ij})}{k}\right)}^{2}}.$$

The average success rate SR(k) quantifies the model’s ability to correctly identify at least one TP in the top k patients. It is defined as


(10) $$SR(k)=\frac{1}{N}\sum_{i=1}^{N}I(\cup_{j=1}^{k}{\text{TP}}_{ij}),$$

where N is the total number of patient records, and I(…) is the indicator function that equals 1 if the union of TPs in the top k predictions for the i-th index patient is not empty and 0 otherwise. The union $\cup_{j=1}^{k}{\text{TP}}_{ij}$ combines all the TPs across the top *k* for the *i*-th index patient. To quantify the variability in binary success outcomes, the empirical standard deviation $s_{k}\;$of the SR was calculated.

A grid search was conducted to find the optimal parameters for vectorization and the concept similarity threshold.^40^ The performance of each parameter set was evaluated based on the average precision for the most similar patient. The parameters that varied during the grid search included the following:

- *binary* (whether to use binary term frequencies): [True, False].
- *max_df* (maximum document frequency for terms): [1.0, 0.9, 0.8, 0.6, 0.4].
- *min_df* (minimum document frequency for terms): [1, 2, 5, 8, 10].
- *threshold* θ (similarity threshold for concept embeddings): [0.8, 0.85, 0.9, 0.95].

*Binary* weighting denotes the presence or absence of a concept without considering its frequency. A *max_df* of 0.9 means that concepts that occur in more than 90% of the documents are ignored. A *min_df* of 5 means that concepts that appear in fewer than 5 documents are ignored.

The evaluation was performed separately for prevalent and infrequent diagnoses in the dataset:

- Prevalent: Primary diagnoses that occur more than 10 times.
- Infrequent: Primary diagnoses that occur between 2 and 10 times.

“Prevalent” and “Infrequent” refer to the occurrences in the dataset and not to the actual prevalence in the population. This procedure was crucial to avoid distorting the results for precision and to evaluate the effectiveness of the model in the context of rare diseases, where data scarcity is a major challenge. For infrequent diagnoses, only the most similar patient was identified and evaluated.

In a subsequent stage, rather than selecting the most similar patient based on cosine similarity, patients were selected at random for validation. This approach was employed to assess the robustness of the model by comparing the SR and precision scores obtained from random case selection against those from similarity-based selection.

### Additional evaluations

For the clinical interpretation of the results, patients were grouped according to their primary diagnoses. The summed TF-IDF scores (without semantic vector embeddings) of the concepts were calculated for each group, thereby demonstrating the relative importance of the concepts within the context of each disease group. The high-scoring concepts are given greater weight in the similarity analysis, enhancing the precision of patient comparisons. The 5 concepts with the highest scores were identified. Three medical experts from the departments of gastroenterology, pulmonology and endocrinology at the University Hospital in Frankfurt assessed the clinical relevance of these concepts for the respective diseases. A relevance rating on a scale of 1 to 5 (with 1 indicating a low level and 5 indicating a high level of relevance) was assigned by the experts to each of the top 5 concepts related to a disease. The mean and standard deviation (SD) were then calculated.

*t*-distributed stochastic neighbour embedding (*t*-SNE) is a dimensionality reduction technique that projects high-dimensional data onto a 2-dimensional plane while preserving local relationships.^41^ This approach was utilized for the visualization of TF-IDF weighted patient records, with the objective of providing insights into the relationships between them. To simplify the visualization, only records with a single primary diagnosis were considered. To ascertain the efficacy of the TF-IDF weights in discerning data at varying degrees of predictive granularity, the visualization and analysis techniques were extended to encompass distinct levels of ICD-10 coding.

### Implementation

The implementation was conducted using Python version 3.11.5, in conjunction with the Averbis Health Discovery tool version 6.18.1 for NLP tasks. The database management was handled using PostgreSQL version 16.1. Furthermore, the data were standardized and managed according to the OMOP CDM version 5.3. The Sklearn library was employed to calculate the weights, cosine similarity and *t*-SNE.^42^ Furthermore, the “*GanjinZero/coder_all*” model from the multilingual checkpoint was utilized for the generation of knowledge-based semantic embeddings, which demonstrated superior performance compared with other embedding methods.^14^^,^^43^ Several engineering experts were consulted regarding the implementation of the project and were involved in the repetition of the analyses, with the objective of ensuring the reproducibility of the results. The study was approved by the Ethics Committee of the Medical Faculty of Goethe University Frankfurt (reference number: 2022-1088).

## Results

### Data analysis

**Figure 2.**
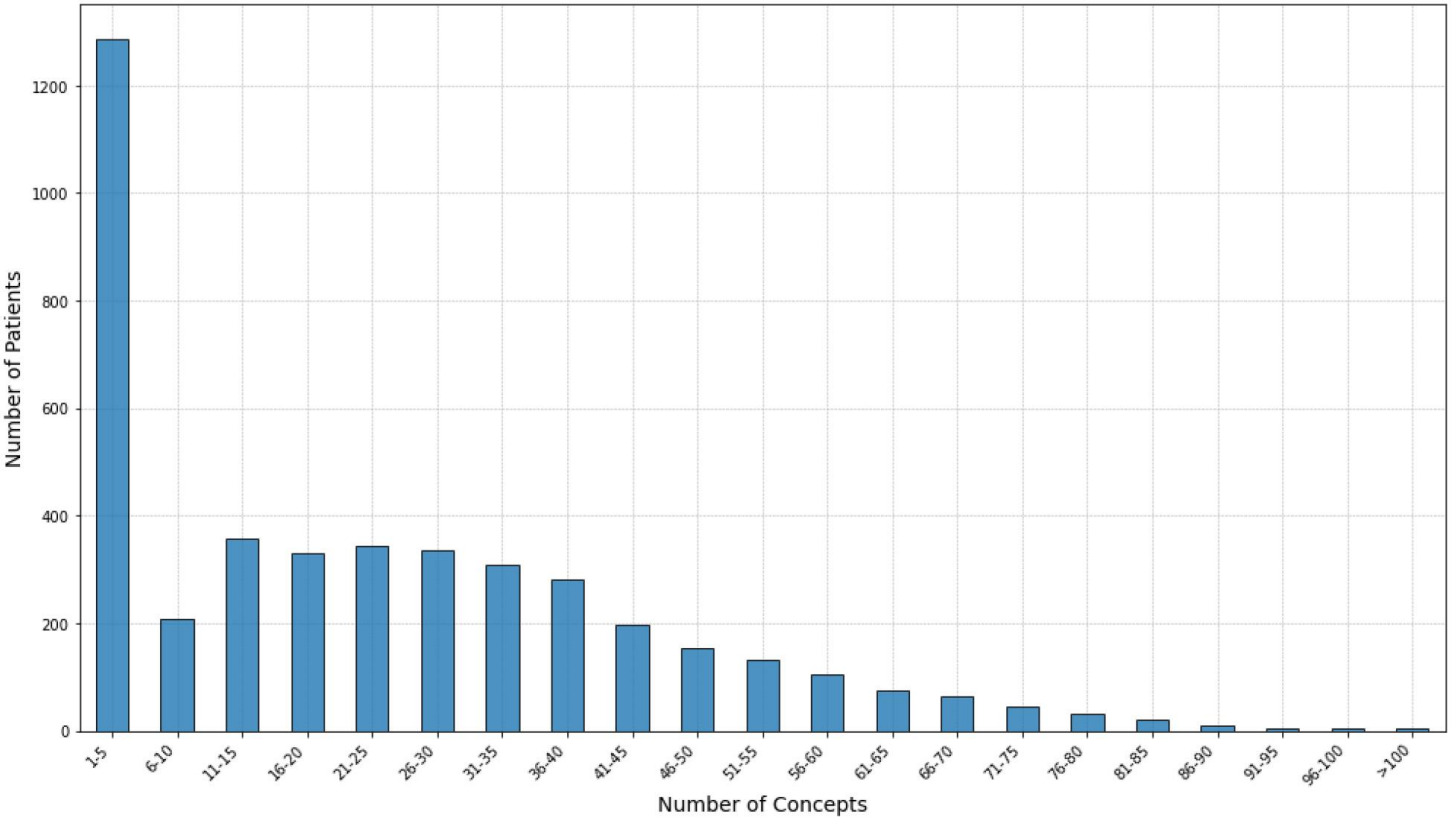
The frequency of patients according to the range of concepts assigned after excluding primary diagnoses and directly indicative concepts. The patient record with the highest number has 127 total concepts and 109 distinct concepts.

The concept frequencies for all 4295 patient records (after exclusion of the primary diagnoses and the directly indicative concepts) are shown in Figure 2. A total of 759 patients had fewer than 2 concepts in their records. The remaining 3536 patient records were the subject of further analysis.

Table 3 provides an overview of the vocabularies used in the OMOP CDM and the frequencies of their concepts indexed in the 3536 patient records. Relation to the reference range is reported for 35 031 of 48 399 (72%) of the total laboratory concepts.

**Table 3.** Vocabularies used in the OMOP CDM and the number of distinct and total medical concepts indexed in the dataset.

Vocabulary	Definition	Distinct concepts	Total concepts
Cancer modifier	Observations/findings/attributes about a cancer	15	1364
HPO	Human Phenotype Ontology^27^	66	12 190
ICD-10-CM	International Classification of Diseases, 10th Revision, Clinical Modification in the United States of America^44^	48	223
ICD-10-GM	International Statistical Classification of Diseases and Related Health Problems, 10th Revision, German Modification^17^	324	3823
LOINC	Logical Observation Identifiers Names and Codes^10^	40	48 399
OMOP extension	Created by OMOP (contains many COVID-19 relevant concepts)	4	1943
SNOMED	Systematized Nomenclature of Medicine^30^	1557	28 369
**Total**		**2054**	**96 311**

The distinct concepts are those that are discernible from one another, while the total value represents the frequency of all the concepts within a given vocabulary.

A total of 2728 records (77%) have 1 primary diagnosis, 668 records (19%) have 2 primary diagnoses, 117 records (3%) have 3 primary diagnoses, 23 records (1%) have 4 primary diagnoses, and 1 record has 5 primary diagnoses. Patients with multiple diagnoses often have several related conditions. For example, a patient may have the following set of diagnoses: “D86.9, *Sarcoidosis*”; “D86.0, *Pulmonary sarcoidosis*”; “A15.1, *Tuberculosis of lung, confirmed by culture only*”; and “A15.0, *Tuberculosis of lung, confirmed by sputum microscopy with or without culture.*”

There are 48 different primary diagnoses in the patient records. Among these, 30 diagnoses (63%) are prevalent, occurring in more than 10 records; 12 diagnoses (25%) are infrequent, appearing in 2 to 10 records, and 6 diagnoses (13%) are observed only once.

### Evaluation results

Table 4 shows that the “Random” method, which serves as the baseline, has the lowest performance across all the metrics. The “TF” method improves significantly over the baseline method and achieves an SR of 0.89 in the top 10, meaning that for 89% of the index patients with prevalent diagnoses, at least one of the 10 most similar patients had a TP match with the index patient's primary diagnoses. For infrequent diagnoses, the SR is 0.20. The “TF-IDF” method shows further enhancements, with an SR of 0.91 in the top 10 for prevalent diagnoses, and an SR of 0.14 in the most similar patient for infrequent diagnoses. The “TF-IDF with Semantic Vector Embeddings” method delivers strong overall performance. With respect to the threshold value of 0.95, a total of 673 concepts were replaced via concept mapping. For index patients with prevalent diagnoses, the method returned a precision of 0.48 for the most similar patient and 0.43 for the 10 most similar patients. For index patients with infrequent diagnoses, a precision of 0.18 was achieved. The results suggest that while the various methods effectively identify a substantial number of TPs, they concomitantly retrieve an even larger proportion of FPs. For diagnoses that are infrequent, the number of FPs is especially pronounced.

**Table 4.** Performance comparison of different methods in predicting primary diagnoses: random, TF, TF-IDF, and TF-IDF with semantic vector embeddings.

Method	**Metric** ($s_{k}$)	Prevalent diagnoses				Infrequent diagnoses
		Top 1	Top 3	Top 5	Top 10	Top 1
Random	SR	0.17 (0.38)	0.39 (0.49)	0.52 (0.50)	0.68 (0.47)	0.04 (0.20)
	Precision	0.17 (0.38)	0.17 (0.24)	0.17 (0.20)	0.17 (0.17)	0.04 (0.38)
TF {“binary”: True, “max_df”: 0.6, “min_df”: 10}	SR	0.47 (0.50)	0.73 (0.44)	0.82 (0.38)	0.89 (0.31)	**0.2 (0.40)**
	Precision	0.47 (0.50)	0.43 (0.34)	0.42 (0.30)	0.41 (0.27)	**0.2 (0.40)**
TF-IDF {“binary”: True, “max_df”: 0.6, “min_df”: 8}	SR	0.47 (0.50)	**0.74 (0.44)**	**0.83 (0.38)**	**0.91 (0.29)**	0.14 (0.35)
	Precision	0.47 (0.50)	0.45 (0.35)	0.44 (0.31)	**0.43 (0.27)**	0.14 (0.35)
TF-IDF with semantic vector {“binary”: False, “max_df”: 1.0, “min_df”: 8, “θ”: 0.95}	SR	**0.48 (0.50)**	**0.74 (0.44)**	0.82 (0.38)	0.89 (0.31)	0.18 (0.38)
	Precision	**0.48 (0.50)**	**0.46 (0.35)**	**0.45 (0.32)**	0.43 (0.28)	0.18 (0.39)

The table includes success rates (SR) and precision metrics (with empirical standard deviation $s_{k}$) for prevalent diagnoses (Top 1, Top 3, Top 5, and Top 10) and infrequent diagnoses (Top 1). The hyperparameters used are shown in curly brackets. Owing to the definition of their calculation, the precision and success rate are always identical for the Top 1 category. The highest values are marked in bold.

The results of the expert evaluation, in terms of the relevance of the high-weighted concepts for the respective diseases, yielded similar ratings across all medical specialties. The mean relevance rating for the concepts of endocrinological diseases is 3.2 (SD = 1.0), whereas the mean rating for the concepts of gastroenterological diseases is 2.6 (SD = 0.9). The mean relevance rating for the concepts of pneumological diseases is 2.1 (SD = 1.2). The detailed ratings are available in File S1.

**Figure 3.**
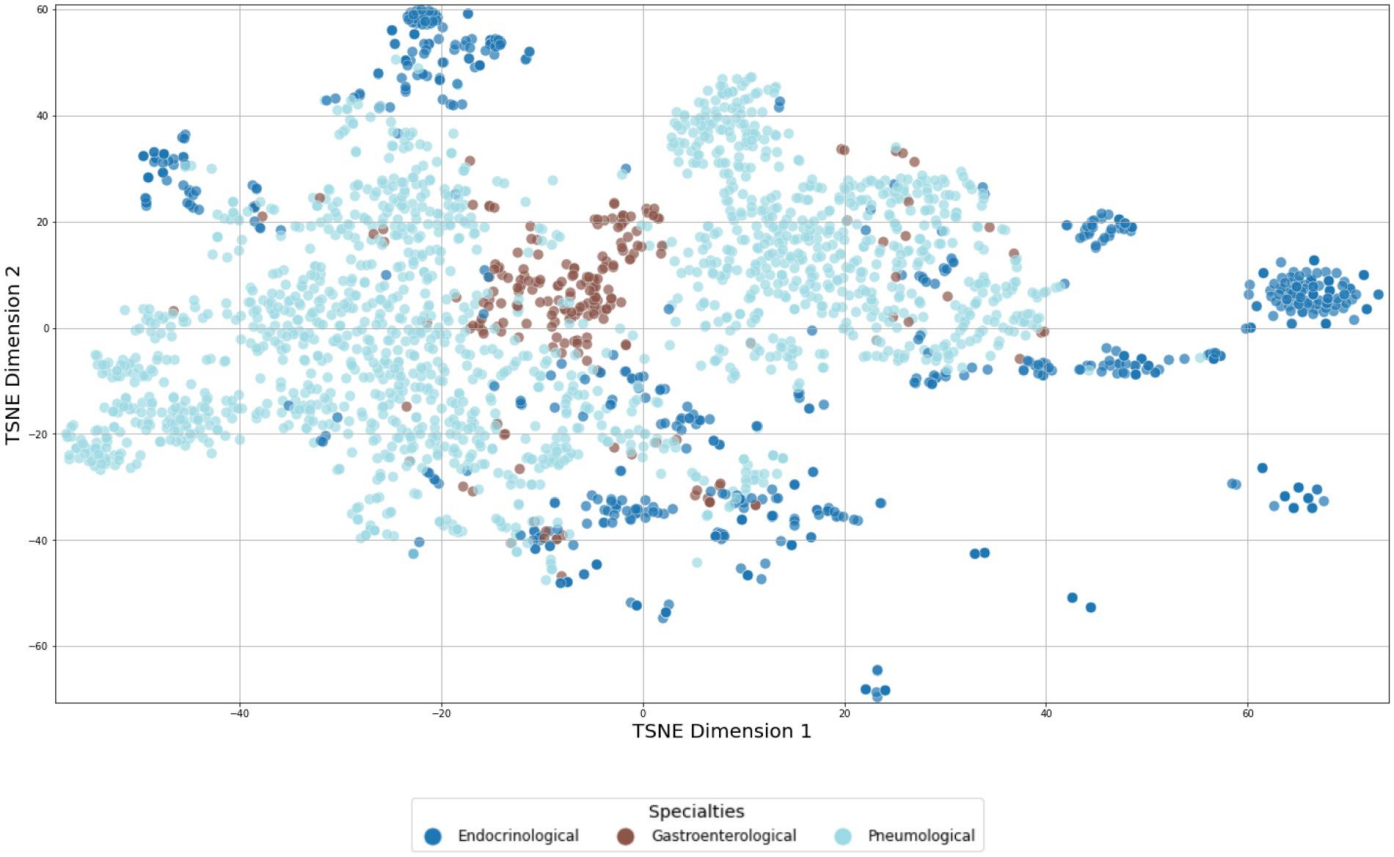
*t*-SNE visualization of patient records represented by TF-IDF vectors, categorized by medical specialties (Endocrinological, Gastroenterological, and Pneumological). Each color represents a different medical specialty.

**Figure 4.**
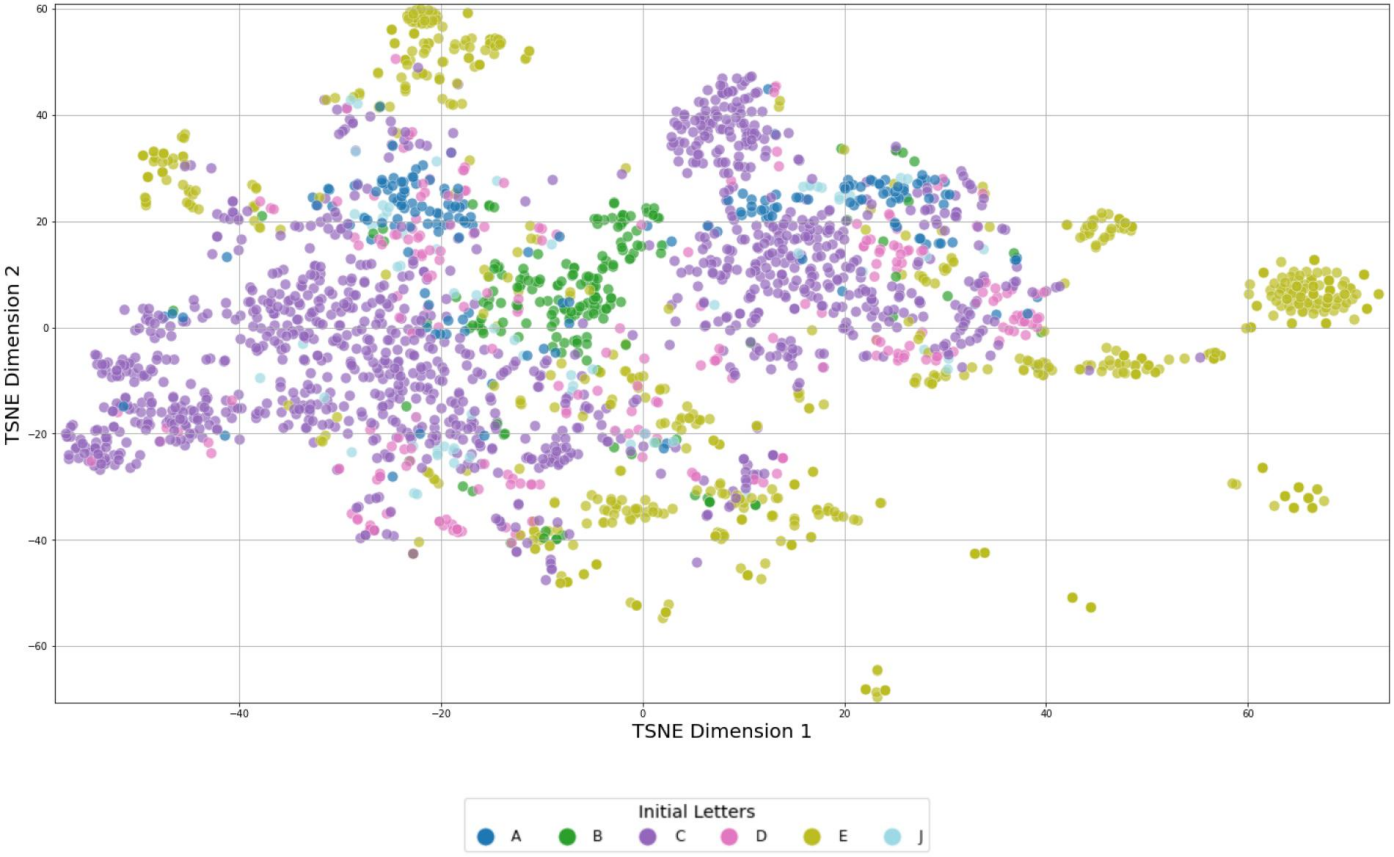
*t*-SNE visualization of patient records represented by TF-IDF vectors, categorized by the initial letter of their primary diagnosis codes. Each color represents a different initial letter. In ICD-10 coding, the initial letter indicates the chapter, representing a broad category of diseases, such as “A” for certain infections.

**Figure 5.**
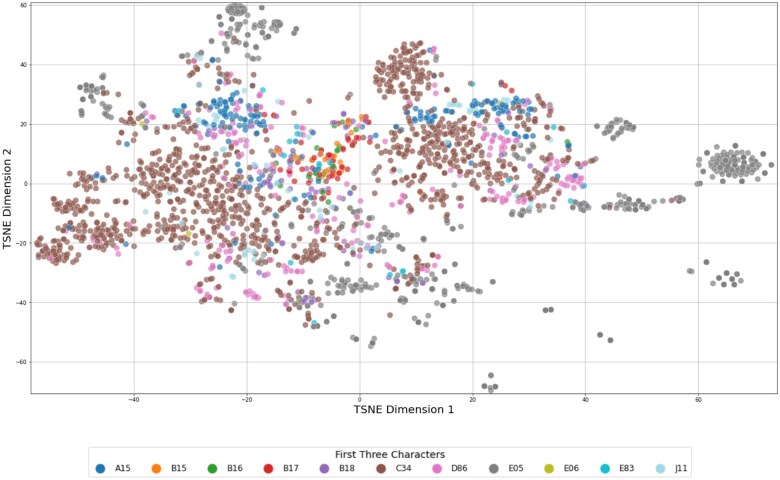
*t*-SNE visualization of patient records represented by TF-IDF vectors, which are based on the first 3 characters of their primary diagnosis codes. Each color represents a different prefix. The first 3 characters in the ICD-10 codes represent the category of the disease, providing a more specific classification than the initial letter alone. For example, “A15” indicates respiratory tuberculosis.

**Figure 6.**
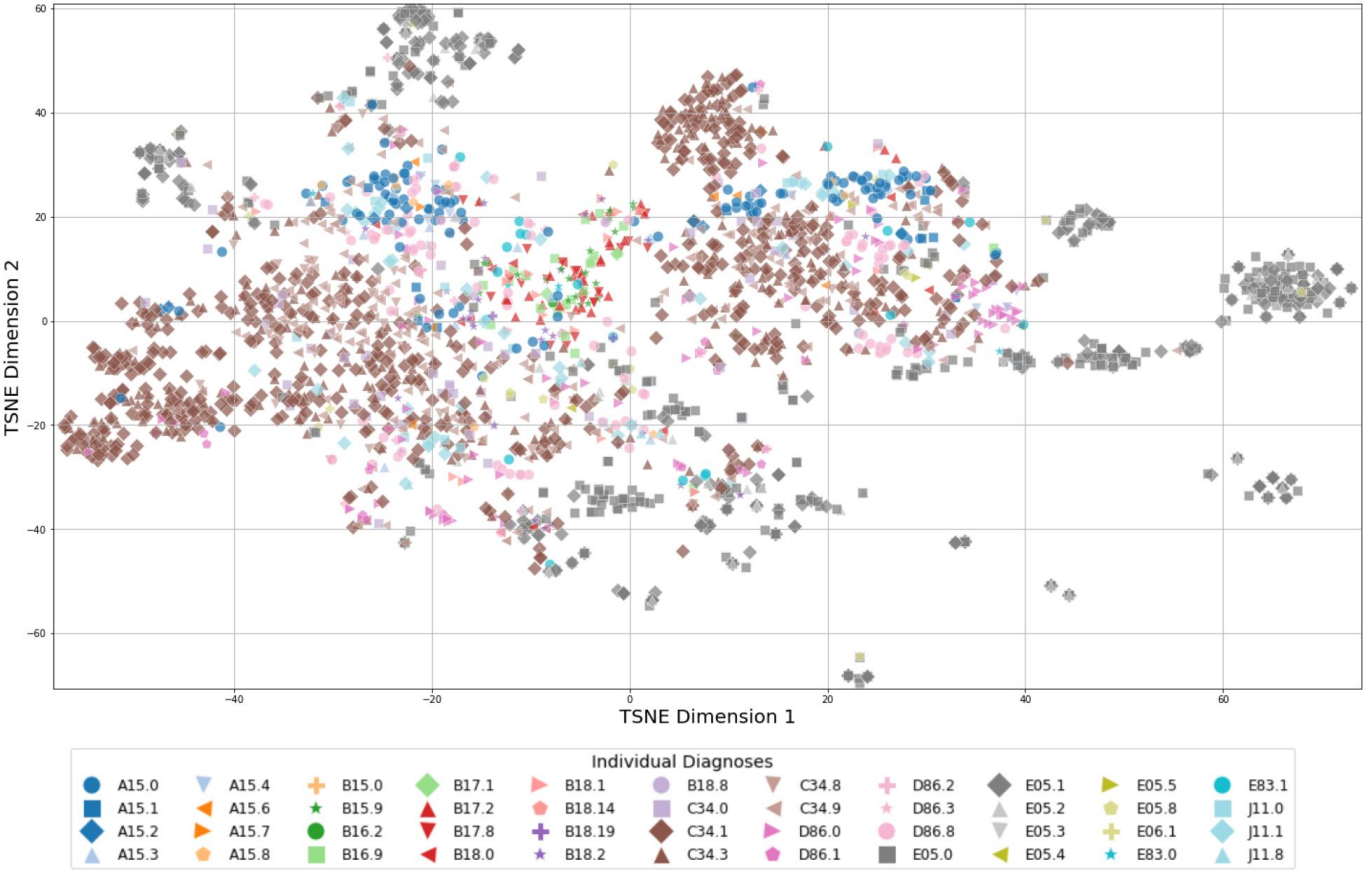
*t*-SNE visualization of patient records represented by TF-IDF vectors, displayed individually. Each color and marker represents a unique primary diagnosis (ICD-10).

Figures 3-6 illustrate the results of the *t*-SNE analysis, which is based on TF-IDF weighted vectors, highlighting the distribution of patient records. The color-coding employed in the figures was implemented subsequent to the t-SNE computation, with the objective of visually representing the degree of similarity among categories of diagnosis codes at varying levels of ICD-10 granularity. This visual aid is designed to illustrate the performance metrics presented in Table 5. With increasing granularity of the ICD-10 coding, as shown in Figures 5 and 6, fewer distinct groups and fewer larger patterns can be seen in the data.

**Table 5.** Performance comparison of TF-IDF in predicting primary diagnoses across various levels of granularity: Specialties, Initial Letters, First 3 Characters, and Individual Diagnoses.

Method	**Metric** ($s_{k}$)	Prevalent diagnoses				Infrequent diagnoses
		Top 1	Top 3	Top 5	Top 10	Top 1
Specialities	SR	0.94 (0.24)	0.98 (0.14)	0.99 (0.10)	0.99 (0.10)	0.85 (0.36)
	Precision	0.94 (0.24)	0.93 (0.20)	0.92 (0.19)	0.91 (0.19)	0.84 (0.36)
Initial Letters	SR	0.85 (0.36)	0.94 (0.24)	0.96 (0.20)	0.98 (0.14)	0.57 (0.50)
	Precision	0.85 (0.35)	0.83 (0.29)	0.82 (0.27)	0.81 (0.26)	0.57 (0.50)
First 3 Characters	SR	0.83 (0.38)	0.92 (0.27)	0.95 (0.22)	0.98 (0.14)	0.45 (0.50)
	Precision	0.83 (0.38)	0.81 (0.31)	0.79 (0.30)	0.78 (0.29)	0.45 (0.50)
Individual Diagnoses	SR	0.47 (0.50)	0.74 (0.44)	0.83 (0.38)	0.91 (0.29)	0.14 (0.35)
	Precision	0.47 (0.50)	0.45 (0.35)	0.44 (0.31)	0.43 (0.27)	0.14 (0.35)

The table includes success rates (SR) and precision metrics (with empirical standard deviation $s_{k}$) for prevalent diagnoses (Top 1, Top 3, Top 5, and Top 10) and infrequent diagnoses (Top 1). The results for the individual diagnosis are the same as those for the TF-IDF values in Table 4 and are used here as a benchmark.

## Discussion

The study analyzed 4295 patient records, focusing on 3536 records after excluding primary diagnoses and directly indicative concepts. Most patients had a single primary diagnosis, with fewer patients having multiple related conditions. There were 48 different primary diagnoses. The performance comparisons of the diagnosis prediction methods revealed that while advanced methods such as TF-IDF with semantic vector embeddings showed higher precision and success rates, the differences between the methods were not significant when considering empirical standard deviations.

The average success rate of 0.91 achieved with the TF-IDF method for the 10 most similar patients demonstrates that correct diagnoses are frequently identified. However, the considerably lower average precision of 0.43 indicates that, on average, the number of FPs exceeds that of TPs. This discrepancy underscores the necessity for caution, as the high rate of FPs has the potential to compromise the practical utility of the method in diagnostic decision support systems. In particular, a high FP rate could increase the cognitive burden on clinicians by introducing irrelevant or misleading suggestions. Nevertheless, the findings suggest that the correct diagnosis is present in 91% of instances and on average, 43% of the 10 most similar cases contain the correct diagnosis, offering physicians a significant subset of pertinent results. In the context of rare diseases, where early detection is imperative, it is precisely this awareness of the right condition that could be the crucial tool to identify a disease that might otherwise have been overlooked.

The precision and success rate are lower for infrequent diagnoses, indicating that the model struggles to identify the TPs due to an imbalanced case base. Conversely, in cases of frequent diagnoses, a greater number of analogous cases are available in the case base, thereby increasing the probability of identifying a TP for the most similar patient. This underscores the inherent challenge of supporting rare diagnoses in a system where case representation is imbalanced, emphasizing the necessity for targeted strategies to enhance the model's performance for underrepresented conditions. The underrepresentation of gender-specific patterns, in conjunction with the subsequent biases in similarity calculations, has the potential to further diminish the system's capacity to accurately identify TPs.

The expert evaluation regarding the relevance of the high-weighted concepts for the respective diseases resulted in an average concept relevance of 2.6 across all medical specialties. This suggests that the TF-IDF approach identifies key concepts for similarity retrieval that may not always align with the key indicators that physicians consider most relevant for diagnosing these diseases.

The *t*-SNE visualizations demonstrated that as the granularity of ICD-10 coding increased, the number of distinct groups in the data decreased, resulting in a decline in prediction precision. While it may seem intuitive that lower levels of granularity (ie, less specific diagnosis codes) are easier to predict due to their broader scope, the objective of this analysis was to quantify the exact differences in precision and success rates at varying granularity levels. The finding that using only the first 3 characters of the ICD-10 code nearly doubles the precision compared with using the full ICD-10 code is particularly noteworthy. Especially because in the context of complex rare disease patterns, the automated determination of a definitive ICD-10 diagnosis is of secondary importance. Therefore, the utility of such a system as a support system should be prioritized over its use as a standalone diagnostic tool. The provision of broader ICD-10 codes could already enable healthcare professionals, particularly in rural areas with limited experience in such cases, to gain initial insight into the disease process, facilitating the initiation of appropriate and timely treatment, further clarification through clinical guidelines, or referrals to experts in the field. In any case, the diagnosis of a rare disease necessitates a series of specialized examinations, genetic tests, and assessments by experts to be confirmed.

Several limitations must be acknowledged to provide a comprehensive understanding of the findings and their implications. The granularity of laboratory values falls short, as categorizing results simply as below, within, or above the reference range does not capture the nuances needed for clinical decision-making.^45^ The medical experts involved in this study agreed that someone significantly above the normal range should be assessed differently from someone only slightly above it. However, the establishment of a more refined categorization, encompassing the delineation between borderline and significantly elevated results, necessitates the implementation of further threshold definitions. The definition of threshold values for many laboratory parameters is subject to debate, as clinical significance often depends on context, reference ranges, and individual patient characteristics. This process is frequently subjective and varies across clinical guidelines. A study by Albers et al. underscores the prevalence of bias in laboratory data within EHRs, often resulting from the clinical context in which it is collected. This bias can be obscured by simplistic summaries such as the means or standard deviations, hindering the discernment of clinically significant patterns. Their proposed algorithm demonstrates how model-based summarization can capture more informative, interpretable, and robust representations of sparse and irregular laboratory data for downstream phenotyping tasks.^46^

LOINC codes play a crucial role in ensuring the interoperability of laboratory results across different healthcare systems. However, they also present significant challenges. Real-world experience shows that these codes are not uniformly assigned across laboratories, complicating the interpretation of results. For example, 2 tests with the same LOINC code might not yield equivalent results due to differences in how the tests are conducted and interpreted.^12^ This inconsistency is further exacerbated by the lack of a computable infrastructure within LOINC to enable logical inferences and relationships between parts, terms, and concepts.^11^ To summarize, the codes are often not assigned correctly, and even when they are assigned, they are not always comparable, which is a general problem with the use of LOINC codes. At present, there are no alternative interoperability solutions to LOINC.

While the integration of semantic embeddings aims to address some of these standardization challenges, it introduces its own set of issues. The use of semantic embeddings can be seen as deviating from traditional standards, potentially undermining the very interoperability they seek to enhance. The difference in performance between similarity analysis with and without semantic embeddings might not be substantial since concepts have already undergone a level of standardization. This calls into question the added value of semantic embeddings, especially given the increased processing time they require, which can be a limiting factor in real-world applications.

In this study, only patients aged 18 and older were considered, which may limit the generalizability of the findings to younger populations who suffer from rare diseases, potentially overlooking unique diagnostic challenges in pediatric cases. This is particularly significant as many rare diseases are diagnosed in early childhood.^47^ Furthermore, incorporating the chronological sequence of events and time-sensitive embeddings could enhance the model's capacity to reflect the progression of the disease.^48^

**Figure 7.**
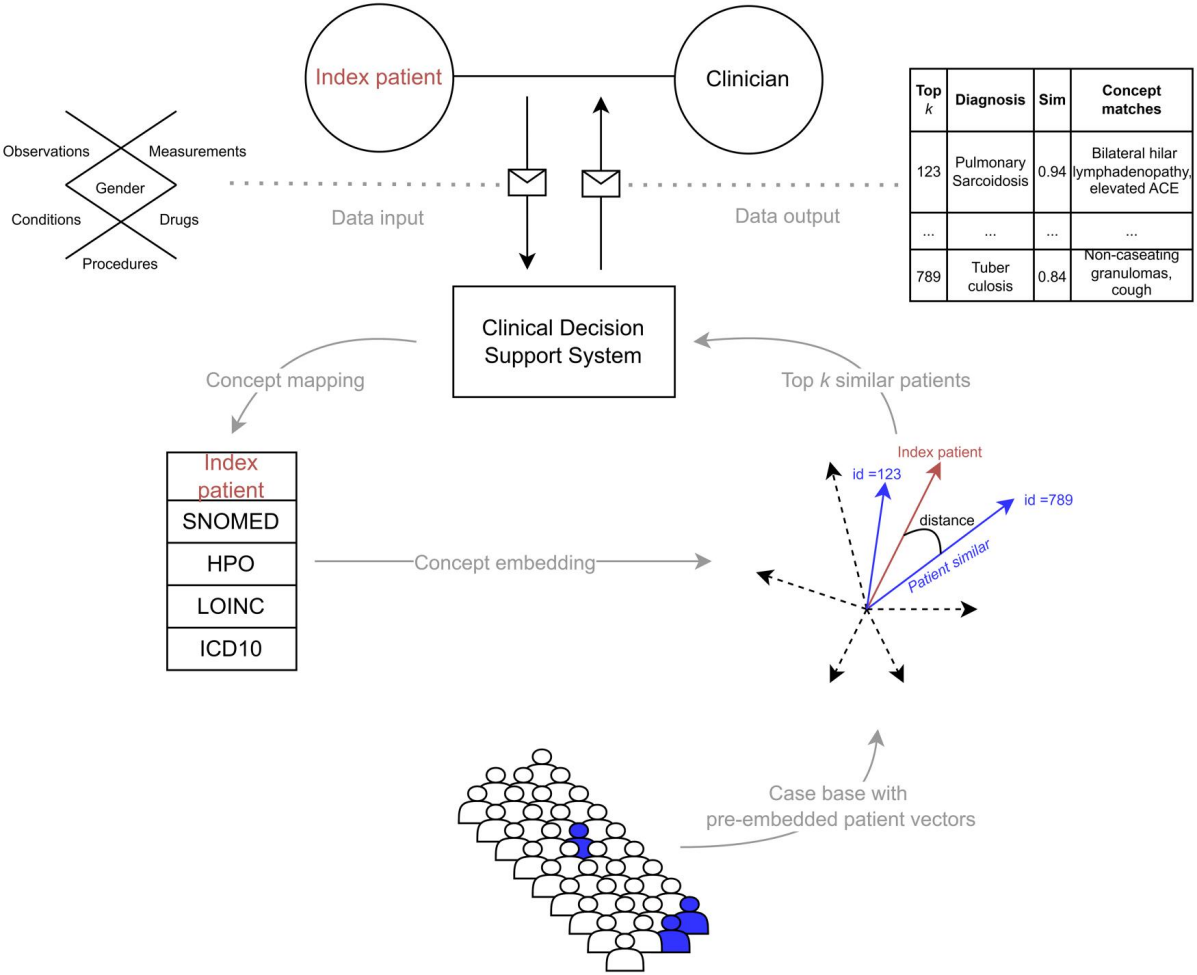
The clinical decision support system employs a comparison of the index patient to a case base of pre-embedded patients, identifies the top *k* most similar cases, and provides diagnoses, similarity scores, and matched concepts to assist the physician. In addition, the full case details of similar patients can be retrieved.

Despite these limitations and challenges, the CBR approach has demonstrated promising results and is intended for use in a clinical decision support system within the context of a larger project entitled SATURN (“Smart physician portal for patients with unclear disease”).^49^ The decisive factor here is the consideration of user requirements^50^ and a user-centered design of the application,^51^ in which the transparency of the analysis results plays a major role. The next phase of this study involves the integration of patient records from other hospitals in Germany. This will be achieved by harmonizing the data via the OMOP CDM. An overview of the concept-based patient similarity approach implemented in the clinical decision support system is shown in Figure 7.

Future research could investigate the potential benefits of combining CBR with deep learning, with the aim of developing Explainable AI (XAI) solutions. These systems offer the chance of providing information on factual, counterfactual, and semi-factual explanations. Factual explanations describe the actual outcomes based on past cases, counterfactual explanations explore what the outcomes would have been if certain conditions were different, and semi-factual explanations consider what could have happened if some elements remained the same while others changed.^52^

Furthermore, additional research could examine which patient attributes most significantly influence precision to refine the model further and to avoid the curse of dimensionality. As the number of features increases, the model's ability to generalize from the training data becomes more challenging, emphasizing the need for dimensionality reduction techniques or feature selection methods.^53^

## Conclusion

The CBR framework facilitates informed decision-making by enabling the visualization and comparison of reference cases. This approach fosters transparency, enhances physicians' confidence, and is particularly well-suited for rare clinical scenarios. The implementation of varied levels of interoperability, ranging from technical to semantic standards, facilitates the optimization of data sharing and exchange. While CBR demonstrates potential for the diagnostics of rare diseases, its utility is contingent upon the requirements of the decision support system and its intended clinical application.

## Supplementary Material

ocaf092_Supplementary_Data

## Data Availability

The data on which this article is based cannot be made public due to the potential for identification of individuals, given that the data may contain information relating to their health.
