# Supplementary material for: Enhancing diagnostic precision for rare diseases using case-based reasoning
Source: J Am Med Inform Assoc. 2025 Jun 17;33(1):98–111. doi: 10.1093/jamia/ocaf092 (PMC12758460; doi:10.1093/jamia/ocaf092)
Supplement: ocaf092_Supplementary_Data [file ocaf092_supplementary_data.zip › SupplementaryFileA.docx]

**Supplementary File A**

*Table S1:* *The table summarizes the top 5 concepts identified by TF-IDF weights for predicting each disease within the specialties. The relevance rating scale ranges from 1 to 5, with 5 indicating high relevance. The assessments were conducted by a medical expert in each specialty who rated the relevance of the five most important concepts for each primary diagnosis. The German concept names in the table are the translated terms from the Human Phenotype Ontology (HPO) that could be identified in the patients' medical records. In the comment’s column, the physicians could provide specific remarks. The rightmost columns display the average rating (Mean) and the standard deviation (SD) of the ratings.* *An '?' in the rating for a concept indicates that the expert does not have a rating for that concept.*

| **Specialty** | **Primary**  **Diagnosis** | **Top 5 Concepts**  **(below, within, above are relations to the reference range)** | **Relevance Rating**  **(1-5)** | **Comments** | **Mean** | **SD** |
| --- | --- | --- | --- | --- | --- | --- |
| Endocrinology | Toxic diffuse goiter | Feminine gender, Masculine gender, Unwohlsein, Measurement of Severe acute respiratory syndrome coronavirus 2 (SARS-CoV-2), Infection screening | 4, 2, 2, 2, 2 | more common in women, feeling unwell is not specific to this condition, Routine infection/COVID screening is not directly associated | 2.4 | 0.8 |
|  | Toxic uninodular goiter | Feminine gender, Masculine gender, Unwohlsein, Measurement of Severe acute respiratory syndrome coronavirus 2 (SARS-CoV-2), Infection screening | 4, 2, 2, 2, 2 | more common in women, feeling unwell is not specific to this condition, Routine infection/COVID screening is not directly associated | 2.4 | 0.8 |
|  | Toxic multinodular goiter | Feminine gender, Masculine gender, Unwohlsein, Measurement of Severe acute respiratory syndrome coronavirus 2 (SARS-CoV-2), Infection screening | 4, 2, 2, 2, 2 | more common in women, feeling unwell is not specific to this condition, Routine infection/COVID screening is not directly associated | 2.4 | 0.8 |
|  | Thyrotoxic crisis | Triiodothyronine (T3) Free [Mass/volume] in Serum or Plasma (above), Thyroxine (T4) free [Mass/volume] in Serum or Plasma (above), Feminine gender, Creatinine [Mass/volume] in Serum or Plasma (below), Emergency hospital admission | 5, 5, 5, 5, 5 |  | 5.0 | 0.0 |
|  | Thyrotoxicosis | Masculine gender, Thyroxine (T4) free [Mass/volume] in Serum or Plasma (above), Triiodothyronine (T3) Free [Mass/volume] in Serum or Plasma (above), Feminine gender, Unwohlsein | 2, 5, 5, 4, 2 | more common in women, feeling unwell is not specific to this condition | 3.6 | 1.4 |
|  | Subacute thyroiditis | Triiodothyronine (T3) Free [Mass/volume] in Serum or Plasma (above), Thyroxine (T4) free [Mass/volume] in Serum or Plasma (above), C reactive protein [Mass/volume] in Serum or Plasma (above), INR in Blood by Coagulation assay, Emergency hospital admission | 5, 5, 4, 2, 2 | INR is not directly related, it typically does not require emergency hospital admission | 3.6 | 1.4 |
|  | Thyrotoxicosis factitia | Thyroxine (T4) free [Mass/volume] in Serum or Plasma (above), Triiodothyronine (T3) Free [Mass/volume] in Serum or Plasma (within), Triiodothyronine (T3) Free [Mass/volume] in Serum or Plasma (below), Obesity, Hypopituitarism | 5, 5, 2, 2, 2 | T3 levels potentially normal or elevated, Obesity and hypopituitarism are not typical features of this condition | 3.2 | 1.5 |
|  | Hyperthyroidism due to ectopic thyroid nodule | Thyrotropin [Units/volume] in Serum or Plasma (below), Measurement of Severe acute respiratory syndrome coronavirus 2 (SARS-CoV-2), Infection screening, Masculine gender, Presence of other cardiac or vascular implants or grafts | 5, 2, 2, 2, 4 | Gender does not directly influence the development of hyperthyroidism due to an ectopic thyroid nodule, Routine infection/COVID screening is not directly associated | 3.0 | 1.3 |
|  |  |  |  | **Average** | **3.2** | **1.0** |
| Gastroenterology | Acute type B viral hepatitis | Ikterus, Bilirubin.direct [Mass/volume] in Serum or Plasma (above), Aspartate aminotransferase [Enzymatic activity/volume] in Serum or Plasma (above), Alanine aminotransferase [Enzymatic activity/volume] in Serum or Plasma (above), Gamma glutamyl transferase [Enzymatic activity/volume] in Serum or Plasma (above) | 4, 3, 3, 3, 3 | N/A | 3.2 | 0.4 |
|  | Chronic viral hepatitis B with hepatitis D | Alanine aminotransferase [Enzymatic activity/volume] in Serum or Plasma (above), C reactive protein [Mass/volume] in Serum or Plasma, Aspartate aminotransferase [Enzymatic activity/volume] in Serum or Plasma (above), Hepatic fibrosis, Masculine gender | 3, 2, 3, 3, 2, | N/A | 2.6 | 0.5 |
|  | Acute viral hepatitis | Bilirubin.direct [Mass/volume] in Serum or Plasma (above), Alanine aminotransferase [Enzymatic activity/volume] in Serum or Plasma (above), Aspartate aminotransferase [Enzymatic activity/volume] in Serum or Plasma (above), Gamma glutamyl transferase [Enzymatic activity/volume] in Serum or Plasma (above), Ikterus | 3, 3, 3, 2, 4 | N/A | 3 | 0.6 |
|  | Chronic viral hepatitis B without delta-agent | Aspartate aminotransferase [Enzymatic activity/volume] in Serum or Plasma (above), Ikterus, Alanine aminotransferase [Enzymatic activity/volume] in Serum or Plasma (above), INR in Blood by Coagulation assay, Abnormal findings diagnostic imaging of liver+biliary tract | 3, 3, 3, 3, 4 | N/A | 3.2 | 0.4 |
|  | Chronic hepatitis C | Cirrhosis of liver, Masculine gender, Feminine gender, Alanine aminotransferase [Enzymatic activity/volume] in Serum or Plasma (above), Aspartate aminotransferase [Enzymatic activity/volume] in Serum or Plasma (above) | 4, 2, 2, 3, 3 | N/A | 2.8 | 0.7 |
|  | Viral hepatitis A without hepatic coma | Bilirubin.direct [Mass/volume] in Serum or Plasma (above), Ceruloplasmin [Mass/volume] in Serum or Plasma (within), Alanine aminotransferase [Enzymatic activity/volume] in Serum or Plasma (above), Alpha 1 antitrypsin [Mass/volume] in Serum or Plasma (within), Aspartate aminotransferase [Enzymatic activity/volume] in Serum or Plasma (above) | 3, 1, 3, 1, 3 | N/A | 2.2 | 1.0 |
|  | Acute hepatitis E | Aspartate aminotransferase [Enzymatic activity/volume] in Serum or Plasma (above), Alanine aminotransferase [Enzymatic activity/volume] in Serum or Plasma (above), Gamma glutamyl transferase [Enzymatic activity/volume] in Serum or Plasma (above), Bilirubin.direct [Mass/volume] in Serum or Plasma (above), Ceruloplasmin [Mass/volume] in Serum or Plasma (within) | 3, 3, 3, 3, 1 | N/A | 2.6 | 0.8 |
|  | Hepatitis D superinfection of hepatitis B carrier | Hepatic failure, Cholinesterase [Enzymatic activity/volume] in Serum or Plasma (below), Jaundice, Aszites, Cholinesterase [Enzymatic activity/volume] in Serum or Plasma (within) | 4, 2, 3, 4, 2 | N/A | 3 | 0.9 |
|  | Hepatic coma due to acute hepatitis B | Jaundice, Hepatorenal syndrome, Residual hemorrhoidal skin tags, Hepatisch Enzephalopathie, Colon diverticulosis without perforation, abscess or indication of bleeding | 3, 3, 2, 4, 1 | N/A | 2.6 | 1.0 |
|  | Chronic viral hepatitis B without Delta virus, phase unspecified | Cirrhosis of liver, Transferrin.carbohydrate deficient/Transferrin.total in Serum or Plasma (above), Transferrin [Mass/volume] in Serum or Plasma (below), Alanine aminotransferase [Enzymatic activity/volume] in Serum or Plasma (above), Arthropathy of multiple joints | 4, 2, 1, 3, 1 | N/A | 2.2 | 1.2 |
|  | Hepatic coma due to viral hepatitis A | Akinetisch-rigides Parkinson-Syndrom, Hepatisch Enzephalopathie, Acute gastric ulcer with hemorrhage, Ceruloplasmin [Mass/volume] in Serum or Plasma (below), Acute gastric ulcer without hemorrhage AND without perforation | 1, 5, 2, 1, 1 | N/A | 2 | 1.6 |
|  | Chronic viral hepatitis | C reactive protein [Mass/volume] in Serum or Plasma, Alanine aminotransferase [Enzymatic activity/volume] in Serum or Plasma (above), Ikterus, INR in Blood by Coagulation assay, Calcium [Moles/volume] in Serum or Plasma (above) | 2, 3, 4, 3, 2 | N/A | 2.8 | 0.8 |
|  | Acute hepatitis C | Copper [Mass/volume] in Serum or Plasma (within), Post hematopoietic stem cell transplant without current immunosuppression, Diffuse large B-cell lymphoma (nodal/systemic with skin involvement), Ikterus, Ceruloplasmin [Mass/volume] in Serum or Plasma (below) | 1, 1, 1, 4, 1 | N/A | 1.6 | 1.2 |
|  | Chronic viral hepatitis B without Delta virus, phase 4 | Human immunodeficiency virus infection, Number of T helper cells in HIV disease: Category 3, Clinical categories of HIV disease: Category C, Beta-2-Microglobulin [Mass/volume] in Serum or Plasma (above), Cholinesterase [Enzymatic activity/volume] in Serum or Plasma (within) | 2, 2, 2, 1, 3 | N/A | 2 | 0.7 |
|  | Acute hepatitis B with delta-agent (coinfection) without hepatic coma | Hepatisch Enzephalopathie, Permanently acquired bleeding disorder, Hepatic failure, Inflammatory disorder of digestive tract, Infection due to resistant bacteria | 4, 2, 5, 2, 3 | N/A | 3.2 | 1.1 |
|  | Hepatic coma due to acute hepatitis B with delta agent | Eosinophilie, Ceruloplasmin [Mass/volume] in Serum or Plasma (below), Low blood pressure, Carcinoembryonic Ag [Mass/volume] in Serum or Plasma (above), Ceruloplasmin [Mass/volume] in Serum or Plasma (within) | 2, 1, 4, 3, 1 | N/A | 2.2 | 1.2 |
|  | Disorder of iron metabolism | Masculine gender, C reactive protein [Mass/volume] in Serum or Plasma, Leukocytes [#/volume] in Blood by Automated count (within), Calcium [Moles/volume] in Serum or Plasma (within), Alanine aminotransferase [Enzymatic activity/volume] in Serum or Plasma (above) | 3, 3, 2, 2, 2 | N/A | 2.4 | 0.4 |
|  | Disorder of copper metabolism | Disease of liver, Masculine gender, C reactive protein [Mass/volume] in Serum or Plasma, Leukocytes [#/volume] in Blood by Automated count (within), Albumin [Mass/volume] in Serum or Plasma (within) | 4, 2, 2, 2, 3 | N/A | 2.6 | 0.8 |
|  | Disorder of plasma protein metabolism | Elsewhere classified hospital-acquired pneumonia that either exists on admission or occurs within 48 hours of admission with known hospitalization up to 28 days ago, Interleukin 6 [Mass/volume] in Serum or Plasma (above), Calcium [Moles/volume] in Serum or Plasma (below), Palliative care, Chronic obstructive pulmonary disease, unspecified: FEV1 unspecified | 2, 3, 2, 4, 3 | N/A | 2.8 | 0.7 |
|  |  |  |  | **Average** | **2.6** | **0.9** |
| Pulmonology | Primary malignant neoplasm of respiratory tract | C reactive protein [Mass/volume] in Serum or Plasma (above), Aspartate aminotransferase [Enzymatic activity/volume] in Serum or Plasma, INR in Blood by Coagulation assay, Lymphocytes [#/volume] in Blood by Automated count (below), Leukocytes [#/volume] in Blood by Automated count (above) | 4, 2, 1, ?, ? | What is it about the INR, which appears almost everywhere but without any indication of a change; was it simply determined for all patients? A connection to the diseases mentioned would be new to me | 2.33 | 1.3 |
|  | Tuberculosis of intrathoracic lymph nodes | Fieber, Infection screening, INR in Blood by Coagulation assay, C reactive protein [Mass/volume] in Serum or Plasma (above), Neutrophils [#/volume] in Blood (within) | 4, 3, 1, 4, 3 | N/A | 3 | 1.1 |
|  | Tuberculosis of lung, confirmed by culture only | Neutrophils [#/volume] in Blood (within), Fieber, Lymphocytes [#/volume] in Blood by Automated count (below), Infection screening, Dyspnoe | 3, 4, 1, 1, 4 | According to the textbook, TB causes neutropenia and lymphocytosis, not lymphopenia, but I am not a TB specialist | 2.6 | 1.4 |
|  | Tuberculosis of lung, confirmed by sputum microscopy with or without culture | Lymphocytes [#/volume] in Blood by Automated count (within), Neutrophils [#/volume] in Blood (within), Lactate dehydrogenase [Enzymatic activity/volume] in Serum or Plasma by Lactate to pyruvate reaction, Aspartate aminotransferase [Enzymatic activity/volume] in Serum or Plasma, Erythrocyte sedimentation rate (above) | 1, 3, 2, 2, 4 | N/A | 2.4 | 1.1 |
|  | Primary malignant neoplasm of lung | INR in Blood by Coagulation assay, Alanine aminotransferase [Enzymatic activity/volume] in Serum or Plasma, Abnormal findings on diagnostic imaging of lung, Calcium [Moles/volume] in Serum or Plasma (within), Creatinine [Mass/volume] in Serum or Plasma (within) | 1, 1, 5, 1, 1 | N/A | 1.8 | 1.7 |
|  | Primary malignant neoplasm of lower lobe, bronchus or lung | INR in Blood by Coagulation assay, Abnormal findings on diagnostic imaging of lung, Alanine aminotransferase [Enzymatic activity/volume] in Serum or Plasma, Leukocytes [#/volume] in Blood by Automated count (within), Creatinine [Mass/volume] in Serum or Plasma (within) | 1, 5, 1, 1, 1 | N/A | 1.8 | 1.7 |
|  | Overlapping malignant neoplasm of bronchus and lung | C reactive protein [Mass/volume] in Serum or Plasma (above), Aspartate aminotransferase [Enzymatic activity/volume] in Serum or Plasma, Leukocytes [#/volume] in Blood by Automated count (above), Lactate dehydrogenase [Enzymatic activity/volume] in Serum or Plasma by Lactate to pyruvate reaction (above), Alanine aminotransferase [Enzymatic activity/volume] in Serum or Plasma | 4, 1, 4, 4, 1 | N/A | 2.8 | 1.5 |
|  | Primary malignant neoplasm of main bronchus | Secondary malignant neoplasm of intrathoracic lymph nodes, C reactive protein [Mass/volume] in Serum or Plasma (above), INR in Blood by Coagulation assay, Leukocytes [#/volume] in Blood by Automated count (above), Calcium [Moles/volume] in Serum or Plasma (within) | 4, 4, 1, 4, 1 | N/A | 2.8 | 1.5 |
|  | Sarcoidosis of lung with sarcoidosis of lymph nodes | Interleukin 2 receptor [Units/volume] in Serum or Plasma (above), Abnormal findings on diagnostic imaging of lung, INR in Blood by Coagulation assay, Creatinine [Mass/volume] in Serum or Plasma (within), Leukocytes [#/volume] in Blood by Automated count (within) | 4, 5, 1, 1, 1 | N/A | 2.4 | 1.9 |
|  | Tuberculous pleurisy, confirmed bacteriologically and histologically | Empyema of pleura, Pleural effusion, Creatinine [Mass/volume] in Serum or Plasma (below), Thyrotropin [Units/volume] in Serum or Plasma (within), C reactive protein [Mass/volume] in Serum or Plasma (above) | 4, 4, 1, 1, 4 | N/A | 2.8 | 1.5 |
|  | Influenza with non-respiratory manifestation | Feminine gender, Influenza caused by seasonal influenza virus, C reactive protein [Mass/volume] in Serum or Plasma (above), Upper respiratory tract infection due to Influenza, INR in Blood by Coagulation assay | 1, ?, 4, ?, 1 | N/A | 2 | 1.4 |
|  | Upper respiratory tract infection due to Influenza | Emergency hospital admission, C reactive protein [Mass/volume] in Serum or Plasma (above), INR in Blood by Coagulation assay, Calcium [Moles/volume] in Serum or Plasma (within), Neutrophils [#/volume] in Blood (within) | 1, 4, 1, 1, 3 | N/A | 2 | 1.1 |
|  | Respiratory tuberculosis, bacteriologically and histologically confirmed | Erythrocyte sedimentation rate (above), Lymphocytes [#/volume] in Blood by Automated count (within), Alpha 2 globulin/Protein.total in Serum or Plasma by Electrophoresis (above), C reactive protein [Mass/volume] in Serum or Plasma (above), Calcium [Moles/volume] in Serum or Plasma (within) | 4, 1, 3, 4, 1 | N/A | 2.6 | 1.4 |
|  | Tuberculosis of lung, confirmed histologically | Lymphocytes [#/volume] in Blood by Automated count (within), Lactate dehydrogenase [Enzymatic activity/volume] in Serum or Plasma by Lactate to pyruvate reaction, Infectious disease carrier, Creatinine [Mass/volume] in Serum or Plasma (below), Albumin [Mass/volume] in Serum or Plasma (within) | 1, 1, 1, 1, 1 | N/A | 1 | 0.0 |
|  | Pneumonia and influenza | Emergency hospital admission, C reactive protein [Mass/volume] in Serum or Plasma (above), INR in Blood by Coagulation assay, Gamma glutamyl transferase [Enzymatic activity/volume] in Serum or Plasma, Alanine aminotransferase [Enzymatic activity/volume] in Serum or Plasma | 1, 4, 1, 1, 1 | N/A | 1.6 | 1.2 |
|  | Pulmonary tuberculosis | Lactate dehydrogenase [Enzymatic activity/volume] in Serum or Plasma by Lactate to pyruvate reaction, Aspartate aminotransferase [Enzymatic activity/volume] in Serum or Plasma, Gamma glutamyl transferase [Enzymatic activity/volume] in Serum or Plasma, Hämoptysen, Alanine aminotransferase [Enzymatic activity/volume] in Serum or Plasma | 1, 1, 1, 1, 1 | N/A | 1 | 0.0 |
|  | Primary respiratory tuberculosis, confirmed bacteriologically and histologically | Number of T helper cells in HIV disease: Category 3, Human immunodeficiency virus infection with secondary clinical infectious disease, Beta-2-Microglobulin [Mass/volume] in Serum or Plasma (above), Cholinesterase [Enzymatic activity/volume] in Serum or Plasma (below), Bilirubin.direct [Mass/volume] in Serum or Plasma | 1, 1, 1, 1, 1 | N/A | 1 | 0.0 |
|  | Sarcoidosis | Interleukin 2 receptor [Units/volume] in Serum or Plasma (above), Interleukin 2 receptor [Units/volume] in Serum or Plasma (within), Thyrotropin [Units/volume] in Serum or Plasma (within), INR in Blood by Coagulation assay, C reactive protein [Mass/volume] in Serum or Plasma | 4, 1, 1, 1, 1 | N/A | 1.6 | 1.3 |
|  | Pulmonary sarcoidosis | Localized enlarged lymph nodes, Masculine gender, Abnormal findings on diagnostic imaging of lung, INR in Blood by Coagulation assay, Calcium [Moles/volume] in Serum or Plasma (within) | 4, 1, 1, 4, 1 | N/A | 2.2 | 1.3 |
|  | Lymph node sarcoidosis | Localized enlarged lymph nodes, Masculine gender, Leukocytes [#/volume] in Blood by Automated count (within), Abnormal findings on diagnostic imaging of lung, Creatinine [Mass/volume] in Serum or Plasma (within) | 4, 1, 1, 4, 1 | N/A | 2.2 | 1.3 |
|  | Cutaneous sarcoidosis | Interleukin 2 receptor [Units/volume] in Serum or Plasma (above), Gamma glutamyl transferase [Enzymatic activity/volume] in Serum or Plasma, C reactive protein [Mass/volume] in Serum or Plasma, vaskulitische Veränderungen, Leukocytes [#/volume] in Blood by Automated count (within) | 4, 1, 1, 3, 1 | N/A | 2 | 1.1 |
|  |  |  |  | **Average** | **2.1** | **1.2** |
